# The Trade-Off Between Sanitizer Resistance and Virulence Genes: Genomic Insights into *E. coli* Adaptation

**DOI:** 10.3390/antibiotics14030291

**Published:** 2025-03-11

**Authors:** Vinicius Silva Castro, Yuri Duarte Porto, Xianqin Yang, Carlos Adam Conte Junior, Eduardo Eustáquio de Souza Figueiredo, Kim Stanford

**Affiliations:** 1 Faculty of Agronomy and Zootechnics, Federal University of Mato Grosso (UFMT), Cuiabá 78060-900, MT, Brazil; viniciuscastro06@gmail.com (V.S.C.); ydporto@ufrrj.br (Y.D.P.); eduardo.figueiredo@ufmt.br (E.E.d.S.F.); 2 Faculty of Nutrition, Federal University of Mato Grosso (UFMT), Cuiabá 78060-900, MT, Brazil; 3 Agriculture and Agri-Food Canada Lacombe Research and Development Centre, 6000 C & E Trail, Lacombe, AB T4L 1W1, Canada; xianqin.yang@agr.gc.ca; 4 Center for Food Analysis (NAL-LADETEC), Department of Biochemistry, Chemistry Institute, Federal University of Rio de Janeiro, Av. Horácio Macedo, Polo de Química, bloco C, 1281-Cidade Universitária, Rio de Janeiro 21941-598, RJ, Brazil; conte@iq.ufrj.br; 5 Department of Biological Sciences, University of Lethbridge, Lethbridge, AB T1K 3M4, Canada

**Keywords:** evolutionary mechanisms, bacterial communities, bacteriophage, foodborne bacteria, sanitizer resistance

## Abstract

Background: *Escherichia coli* is one of the most studied bacteria worldwide due to its genetic plasticity. Recently, in addition to characterizing its pathogenic potential, research has focused on understanding its resistance profile to inhibitory agents, whether these be antibiotics or sanitizers. Objectives: The present study aimed to investigate six of the main serogroups of foodborne infection (O26, O45, O103, O111, O121, and O157) and to understand the dynamics of heterogeneity in resistance to sanitizers derived from quaternary ammonium compounds (QACs) and peracetic acid (PAA) using whole-genome sequencing (WGS). Methods: Twenty-four *E. coli* strains with varied resistance profiles to QACs and PAA were analyzed by WGS using NovaSeq6000 (150 bp Paired End reads). Bioinformatic analyses included genome assembly (Shovill), annotation via Prokka, antimicrobial resistance gene identification using Abricate, and core-genome analysis using Roary. A multifactorial multiple correspondence analysis (MCA) was conducted to explore gene–sanitizer relationships. In addition, a large-scale analysis utilizing the NCBI Pathogen Detection database involved a 2 × 2 chi-square test to examine associations between the presence of *qac* and *stx* genes. Results: The isolates exhibited varying antimicrobial resistance profiles, with O45 and O157 being the most resistant serogroups. In addition, the *qac* gene was identified in only one strain (S22), while four other strains carried the *stx* gene. Through multifactorial multiple correspondence analysis, the results obtained indicated that strains harboring genes encoding Shiga toxin (*stx*) presented profiles that were more likely to be sensitive to QACs. To further confirm these results, we analyzed 393,216 *E. coli* genomes from the NCBI Pathogen Detection database. Our results revealed a significant association (*p* < 0.001) between the presence of *qac* genes and the absence of *stx1*, *stx2*, or both toxin genes. Conclusion: Our findings highlight the complexity of bacterial resistance mechanisms and suggest that non-pathogenic strains may exhibit greater tolerance to QAC sanitizer than those carrying pathogenicity genes, particularly Shiga toxin genes.

## 1. Introduction

With the increase in the human population, there is a growing demand for food. This encourages not only increased food production but also improvements in product quality to minimize losses due to contamination by pathogens [1]. In addition to causing economic losses, products contaminated with foodborne pathogens lead to serious health problems, ranging from impacts on consumer well-being to overburdening public health systems, driven by the care required for gastrointestinal illnesses [1]. An emerging pathogen that has particularly concerned the meat industry is Shiga toxin-producing *Escherichia coli* (STEC), which has been associated with foodborne outbreaks leading to serious infections [2]. This infection is largely caused by the production of Shiga toxins mediated by *stx1* or *stx2* genes, which can lead to cell destruction by interfering with ribosomes and inhibiting protein synthesis [3].

The main STEC serogroups associated with foodborne outbreaks include O26, O45, O103, O111, O121, and O145, which along with O157 are commonly classified as the “top seven” serogroups [4]. Although other serogroups exist, these seven are the most frequently identified in severe disease cases worldwide [5]. In addition to concerns about virulence, antibiotic resistance is also a growing global problem which is exacerbated by the inappropriate use of drugs [6]. Antimicrobial use generates a selective pressure which favors the emergence of resistant strains. This scenario is particularly worrying because the use of antibiotics in the treatment of STEC infections is debated, as sublethal doses of antibiotics may increase the release of Shiga toxins and enhance the severity of the infection [7].

Accordingly, the best overall strategy for STEC is likely to minimize food contamination, since human disease can be serious, and antibiotic treatments may be ineffective. In the food industry, the use of sanitizers has increasingly been adopted as a preventive measure to eliminate these pathogens [8]. However, similarly to antibiotic resistance, the genetic plasticity inherent in bacteria can also lead to resistance to sanitizers. A clear example is the emergence of genes that confer resistance to quaternary ammonium chlorides (QACs), compounds widely used in the food industry to eliminate pathogens [9]. Several studies have warned of increasing tolerance to QACs [10,11]. With this increasing resistance, there may be a need to raise the concentrations of QACs used to kill bacteria. However, higher doses would require more thorough rinsing and would increase the risk of toxic QAC residues contacting food.

In response to QAC toxicity, food processors have increased the use of organic compounds that have low or no toxicity, such as peracetic acid (PAA) [12]. However, increased resistance to organic acids has also been noted, and several genes contributing to acid resistance have been identified [13]. One hypothesis investigated in several studies [14,15] is that bacteria often form biofilms which function as a protective and adhesive network. The use of sanitizers without an efficient mechanical biofilm disruption step may inactivate only the outer layer of the biofilm, leaving viable bacteria in the inner layers which are exposed to sublethal doses of the sanitizer and subsequently develop greater resistance [16].

Furthermore, the presence of different bacterial species within a heterogeneous biofilm further increases resistance to antimicrobial compounds. Strains with resistance to antimicrobials may also enhance the survival of pathogens in a biofilm which lack this resistance [17], and these resistant strains may comprise the outer layers of the biofilm [15]. However, some *E. coli* strains with high resistance to heat and other stresses have been found to be exclusively non-pathogenic. This phenomenon was observed by Zhang and Yang [18], where 706 *E. coli* strains present in the NCBI database were found to have the locus of stress tolerance (tLST), and none of these strains also had *stx1* or *stx2*. Furthermore, several other studies have demonstrated that although commensal strains do not cause direct harm to human health, they can serve as a vector for the dissemination of resistance [19,20,21]. In a study performed by Massella et al. [22], it was found that commensal strains of *E. coli* had great genomic variability, which suggests that the commensal flora could serve as a reservoir of virulence and resistance genes for opportunistic pathogens. Therefore, our hypothesis is that strains which are highly resistant to cellular stress may be less pathogenic as there is a trade-off between virulence and the tolerance of *E. coli* to sanitizers and other stressors.

Based on this hypothesis, the present study investigated the heterogeneity in values of resistance to QACs and PAA in *E. coli* strains isolated from cattle, as obtained in our previous study [23]. Using genomic sequencing tools, we analyzed a group of strains with heterogeneity in resistance to these compounds which belonged to six (O26, O45, O103, O111, O121, and O157) of the “top seven” STEC serogroups.

## 2. Results and Discussion

### 2.1. Determination of Serotype, Antimicrobial Resistance, and Presence of stx and Biofilm Genes

Initial evaluations began with the analysis of the agreement between the serologies determined by PCR and in silico serology using the EcOH database. There was an agreement of 87.5% (21/24) between the results obtained by PCR and whole-genome sequencing (WGS) in the determination of the serogroup (Table 1). This level of agreement can be considered high, especially if compared to some previous studies, which indicated greater heterogeneity between the serogroup detection techniques. The inconsistencies observed in other studies can be attributed to several factors, such as differences in the methodology used, genetic variability in the strains analyzed, and, in some cases, limitations in the available databases. An accurate determination of the serogroup has been important for the diagnosis and monitoring of pathogenic strains of *E. coli*. However, the literature has shown that methods such as PCR can also be susceptible to misclassification [24]. In the present study, three strains presented discrepancies between the PCR and WGS results, two of which were related to serogroup O26 and one to O157. This information is important because these two serogroups are frequently implicated in foodborne outbreaks, making correct identification critical for epidemiological investigations.

Although these discrepancies have been observed, the use of PCR remains a viable alternative for initial serogroup characterization, especially in contexts where speed and cost are limiting factors. However, WGS has a significant advantage in accuracy, especially in contexts where typing and detailing the virulence and resistance characteristics of strains are essential to optimize their control [25].

In addition to serology, although all 24 sequenced strains harbored incomplete or fragmented phages related to *stx* gene encoding, WGS in the present study determined that only four isolates were positive for *stx1*, despite the serogroups evaluated being traditionally associated with STEC. This low presence of the gene encoding Shiga toxin can be explained by a phenomenon already described in previous studies, which report the loss of *stx*-encoding phages during recultivation [26]. Additionally, Castro et al. [24] demonstrated that phages carrying *stx1* may have undergone excision from the bacterial genome, leading to the absence of *stx* in some strains, even though they present other factors frequently associated with STEC. In addition, a study conducted by Senthakumaran et al. [27] investigated the loss of the *stx*-encoding gene and confirmed that *stx* genes can be lost both in vitro and in vivo. The authors emphasized that this loss may be related to genomic rearrangements, though the exact cause remains unclear. This loss of virulence may be directly related to environmental conditions and phage dynamics, suggesting that these factors could also influence other characteristics, such as resistance to sanitizers. Also, PCR analyses of Shiga toxins for these isolates were conducted prior to their exposure to sanitizers in our previous study [23] which may have also affected results.

Subsequently, all strains were analyzed for the presence of genes related to sanitizer resistance using ResFinder (using the desinFinder database), and only the S22 strain presented a gene related to QAC resistance (Table 1). The sanitizer resistance phenotypes of these strains had previously been studied by our group [23], but it is important to emphasize that although there was variation in MIC values among the different strains, QACs and PAA remained effective as the highest resistance values noted were 12.5 and 25 µg/mL, respectively (Table 1). Previous studies have reported a resistance value to QACs of 256 µg/mL [28] and up to 2310 ppm for PAA [29]. However, the variation in resistance profiles in strains collected from the same production environment aroused our interest in further investigating resistance to QACs and PAA.

**Figure 1:**
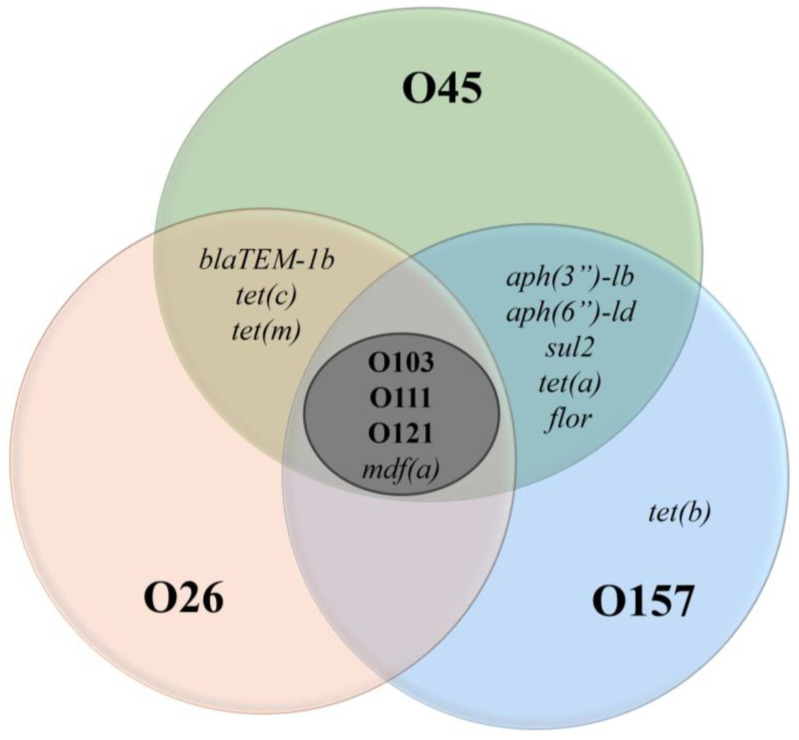
Antimicrobial resistance genes in *E. coli* for six serogroups. Legend: The Venn diagram illustrates the distribution of antimicrobial resistance genes among *E. coli* serogroups O45, O26, O157, O103, O111, and O121. Each circle represents a specific serogroup, and the overlapping areas indicate genes shared between serogroups. The central region (O103, O111, O121) highlights the presence of the *mdf(a)* gene, while the outer regions show unique resistance genes, such as *tet(b)* in O157.

In addition to characteristics related to the presence of *stx1* and the serotype, we also evaluated antimicrobial resistance in the strains (Figure 1). We observed that strains of O103, O111, and O121 presented a low-resistance profile, having only the *mdf(a)* gene. However, although only one gene was detected, it is worth highlighting that *mdf(a)* is responsible for encoding multidrug efflux pump A, which means that it has no specific effect on a molecule itself but acts by expelling a wide range of toxic compounds from the cell, thus conferring broad-spectrum resistance [30,31].

An important point is that *mdf(a)* was identified in all serogroups analyzed, suggesting broad genetic sharing. This dissemination can be explained by its ancient origin [31]. In addition to *mdf(a)*, isolates from serogroups O26, O45, and O157 also had other resistance genes, as illustrated in Figure 1. These additional resistance genes indicate a potential for multidrug resistance and may serve as a warning for possibly difficult-to-treat infections. It is important to highlight that the administration of ineffective drugs can increase the production of Shiga toxin, worsening the clinical condition of patients [32].

Furthermore, it is essential to identify factors promoting the increase in this selective pressure on antimicrobial resistance in specific serogroups. This scenario also reinforces the idea of heterogeneity among serogroups, even when originating from similar sources. Although most studies associate antimicrobial resistance with exposure to antibiotic agents in a set of strains of similar origin, it is still unclear why some serogroups tend to have greater antimicrobial resistance than others. Recent studies suggest that in addition to exposure to antimicrobials, specific genetic factors may be correlated with the development of resistance in each serogroup [33].

**Figure 2:**
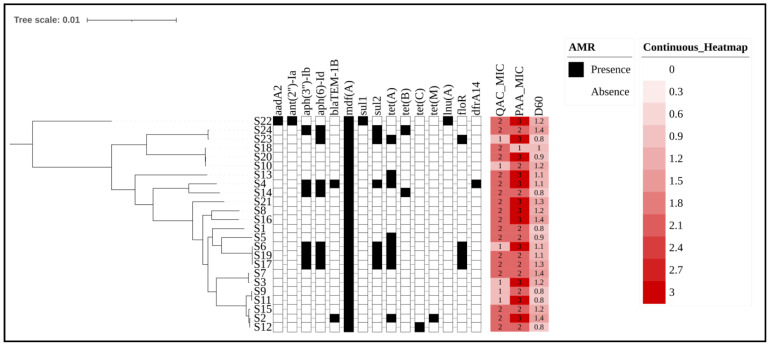
Phylogenetic tree and antimicrobial resistance gene profiles of *E. coli* strains with sanitizer resistance indicators. Legend: The phylogenetic tree illustrates the genetic relationships among *E. coli* isolates from cattle production environments, with branches representing different sequencing strains. The presence or absence of antimicrobial resistance genes is represented by black (present) and white (absent) blocks. Sanitizer resistance levels for QACs and PAA are depicted using a heatmap, where increasing red intensity corresponds to higher resistance. Also, the number inside each block indicates the MIC range for each compound for each strain, with values of 1 (6.25 ppm) 2 (12.50 ppm), and 3 (25 ppm). Finally, D60 represents the bacterial load inactivated after exposure to 60 °C for 1 min and was previously tested in a related study [23]. A lower intensity of color for D60 indicates greater resistance to heat. The numbers inside the blocks represent the logarithmic inactivation load after exposure to heat. The phylogenetic tree was edited using the ITOL website: https://itol.embl.de/ (accessed on 5 February 2025). The Newick tree from core-genome alignment was uploaded, and the metadata were included in binary format (AMR presence/absence) and as a heatmap (sanitizer or D60 value).

As part of the investigation of the relationships between the isolates, we created a phylogenetic tree to assess genetic proximity and cross-reference information for each strain (Figure 2). The phylogenetic tree allowed us to observe that factors related to antimicrobial resistance or phenotypic characteristics, such as the MIC for sanitizers or D value (heat resistance), did not directly influence the genetic proximity between the isolates.

Although phenotypic profiles can vary considerably, genetic relationships transcend small, specific variations in resistance genes. In turn, the phylogenetic tree groups strains with the same serotypes into close clades, regardless of differences in resistance to agents such as QACs or PAA. For example, strains S9 and S11 belong to the same phylogenetic clade, despite presenting distinct profiles for QACs and PAA. This suggests that the genetic relationship between the isolates goes beyond specific phenotypic characteristics, indicating that there is a more conserved genetic basis among the serogroups. Furthermore, factors related to the different seasons of the year when these isolates were collected also did not influence the construction of the phylogenetic tree. However, it is important to highlight that Figure 2 shows the separation of two clusters, which are divided based on the map reference sequence used (EDL-933).

### 2.2. Correlation Between Target Genes and Multiple Correspondence Analysis

Based on the presence and absence of genes among the isolates from the core-genome alignment by Roary, we selected sixteen main target genes related to resistance modulation, biofilm formation, and virulence in which at least one strain differed from the others. The antimicrobial resistance genes were *aph(3)-Ib*, *aph(6)-Id*, *blaTEM-1b*, *sul*, *tet(a)*, *tet(b)*, *tet(c)*, *tet(m)*, and *flor*. Other selected genes were related to metabolism and virulence: *dfra14*, *colrnaI*, *gadA*, *gadB*, *emrE*, *cbtA*, and *stx*. In addition, phenotypic values related to factors such as the D value (heat resistance), biofilm formation, and isolation season of each strain (cold or hot climate) were included (results of previous analyses by Castro et al. [23]). The genes listed above were individually compared in terms of their correlation with QAC and PAA resistance values, and significant relationships are shown in Table 2.

It is worth noting that *stx* and *cbtA* genes presented distinct behaviors, with *stx*, when present, being correlated with lower QAC resistance values, while the presence of *cbtA* was associated with higher PAA tolerance values. Historically, the presence of genes encoding Shiga toxin was originally described in strains of *Shigella dysenteriae* [34]. Furthermore, some studies have shown that lysogenic phages encoding several genes including *stx* led to the evolution and appearance of the first *E. coli* with Shiga toxin [34]. As the inclusion of Shiga toxin genes is mediated by mobile genetic elements, the insertion of *stx* is not conserved. On the contrary, it is a factor amenable to change.

In recent years, several studies have investigated strains of *E. coli* with the inclusion of the locus of stress tolerance (tLST). Furthermore, in a study performed by Zhang and Yang [18], the authors evaluated 18,959 *E. coli* present in the NCBI database for the presence of this locus, and 706 sequences presented an ORF (open reading frame) for tLST or its variants. However, none of the strains evaluated by Zhang and Yang [18] carried a Shiga toxin gene when tLST was present. Possibly, mobile genetic elements may have to compete to occupy restricted spaces in areas where ORFs are present, and these mobile genetic elements may be easily excised by the cell.

One point to be highlighted is that for adaptation to acids, as in the case of PAA, *E. coli* has several mechanisms, such as the *gad* and *rpoS* genes [23,35,36]. Adaptation to acid may be related to the need for strains to remain viable even in adverse environments such as the gastrointestinal tract of cattle. Thus, the presence of the *stx* toxin would have no effect on resistance or sensitivity to a PAA-based sanitizer.

In addition to the negative correlation between *stx* and QACs, the results listed in Table 2 demonstrate a positive relationship between *cbtA* and PAA resistance. The *cbtA* gene is responsible for a toxin–antitoxin system [37]. The *cbtA* toxin interferes with cellular structure and affects microtubules and actin filaments, with a consequent impact on cell division [37]. In a review by Yamaguchi and Inouye [38], the authors discussed the effects of toxin–antitoxin (TA) systems in *E. coli* on the regulation of cell growth and death. An important point is that TA systems have been suggested to be involved in cellular dormancy systems (decrease in cell metabolism) in addition to roles in the modification of cell elongation dynamics. Thus, since PAA is an oxidant of cell walls, proteins, and DNA, it is possible that the *cbtA* gene, when present, may affect cellular survival by modifying the shape of the cell wall, or by decreasing the metabolic response to the sanitizer, potentially implying that PAA has less of an effect in the absence of a cellular response. Accordingly, in a study performed by Tan et al. [39], an overexpression of *cbtA* resulted in a cell growth defect and a loss of rod shape, which, according to our theory, could make the cell even more susceptible to PAA attack. However, more studies need to be performed to clarify the role of *cbtA* in the survival of *E. coli* exposed to a variety of stressors.

**Figure 3:**
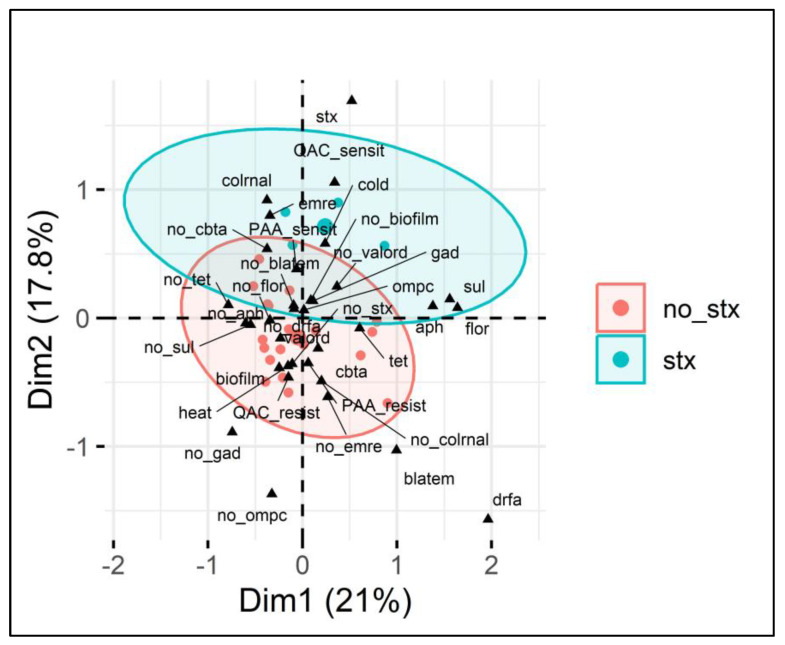
Multiple correspondence analysis (MCA) of *E. coli* sanitizer sensitivity in relation to gene presence. Legend: MCA plot visualizing the relationship between categorical variables (genes and phenotypes) associated with *E. coli* isolates and the presence or absence of the *stx1* gene (indicated by red circles for no_*stx* and blue circles for *stx*). The two dimensions (Dim1 and Dim2) explain 21% and 17.8% of the variance, respectively. The ellipses show clustering based on the presence (blue) or absence (red) of the *stx* gene, highlighting associations between various antimicrobial resistance genes (e.g., *blaTEM*, *sul*, *tet*), sanitizer resistance (PAA and QAC), and other phenotypic traits such as biofilm formation and heat resistance. Key variables such as the presence of *cbtA* and biofilm formation are present within the *stx*-negative group, while the *stx*-positive group shows stronger associations with QAC sensitivity, *colrnaI*, and cold adaptation.

Although this study found a moderate correlation between the presence of two genes and sanitizer resistance, it is known that the effects of individual genes may not be enough to completely explain observed phenotypes. Therefore, we performed a multivariate analysis of genes and phenotypic results to explore associations between multiple variables to understand which groups of genes could collectively influence sanitizer resistance factors. We identified the impact of *stx* presence (based on the correlation data) as a key point to perform multiple correspondence analysis (MCA), and it was possible to group the categories (correlated genes and phenotypes) into two distinct groups (Figure 3).

From Figure 3, it is possible to verify the presence of two separate ellipses categorized based on the presence or absence of the *stx1* gene (no *stx* and *stx*), indicating the contribution of different variables present. Furthermore, the two dimensions (Dim1 and Dim2) explain 21% and 17.8% of the variance, respectively, totaling 38.8%, in the categorical variables analyzed, which is relevant given the complex context of the microbial genome. Similarly to in the analysis of individual genes, the presence of *stx1* was associated with lower QAC resistance, and it also grouped with other genes, such as *colRNA* (controlling colicin production) and *emrE* (encoding efflux pumps). Furthermore, the absence of *stx1* demonstrated that in addition to the positive correlation with the presence of *cbtA* shown in Table 2, the lack of *stx1* grouped with resistance to QAC and PAA in a single cluster, which implies that the presence of the *stx1* gene may make it difficult for a strain to also include tolerance to these sanitizers in its genome.

**Figure 4:**
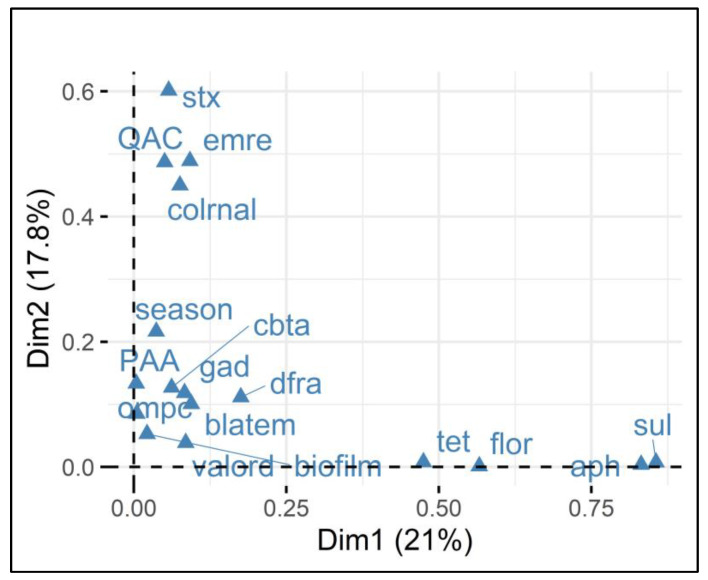
Multiple correspondence analysis (MCA) of *E. coli* resistance and gene presence in relation to sanitizer sensitivity. Legend: The Variable Factor Map demonstrates the grouping (associations) of the analyzed variables associated with *E. coli* isolates and the presence or absence of the *stx1* gene. The two dimensions (Dim1 and Dim2) explain 21% and 17.8% of the variance, respectively. The closer together the points are grouped, the stronger the relationship between the variables. For example, *stx1* and QAC sensitivity, and *emrE* and *colRNAl*.

To increase focus on the relationship between variables, Figure 4 clearly demonstrates the clustering of the analyzed variables that were most closely associated (grouped together) and presents the contribution values in both main dimensions. Thus, the presence of *stx1* was directly associated with a QAC-sensitive phenotype and was clustered with the presence of *emrE* and *colRNA*. Furthermore, resistance to PAA was directly related to several genes, such as *cbtA* (as shown in Table 2), the strain isolation season, *gad* [23], the D value, and the presence of *ompC* and *dfrA*.

Therefore, MCA, when applied to QAC and PAA data, corroborated the information obtained by correlation and provided new insights into the dynamics of resistance to QACs and PAA. Regarding QACs, in addition to the presence of the *stx1* gene conferring greater QAC sensitivity, the grouping showed that the presence of the *emrE* and *colRNAl* genes contributed to reducing sensitivity to QACs. Regarding *emrE*, it is responsible for encoding a multidrug efflux pump that is especially useful for a range of molecules [40]. Therefore, *emrE* may have acted in pumping QACs to the exterior of the cell, attenuating QAC effects on cellular proteins. Colicins coded by *colRNAI* are toxins expressed to suppress competing bacteria and are mediated under stress conditions. Although colicin presence has been attributed to lower resistance to QACs, its participation in QAC resistance is yet undetermined and requires further study.

Regarding PAA resistance, Castro et al. [23] suggested that *gad* genes may have a direct effect on acid resistance. Furthermore, *cbtA* was also present in *E. coli* with increased resistance to PAA. It is worth mentioning that although this relationship between *cbtA* presence and PAA resistance has been observed, there is no mention in the literature of the expression of this gene in response to PAA. Further studies are needed to elucidate the expression of *cbtA* during PAA exposure.

Other genes were grouped nearby, such as *ompC* (a porin that regulates the flow of molecules into the cell [41]). Thus, this regulation may directly limit the permeability of the cell wall to compounds such as PAA, which would make the bacteria more resistant to PAA. Finally, although *dfrA* and *blaTEM* were grouped together with PAA resistance, their effects are related to antimicrobial resistance, such as dihydrofolate reductase inhibition (action of *dfrA* in response to the antibiotic trimethoprim [42]), and the production of beta-lactamases to prevent the action of beta-lactams that act on the cell wall. Therefore, our hypothesis would be that these two genes, although grouped with PAA, more likely represent strains that are capable of greater responses to stress. In fact, PAA tolerance and multidrug resistance were associated in a recent study by Rebelo et al. [43]. The findings demonstrated that *Salmonella* and *Enterococcus faecium* exhibited greater tolerance to sanitizer when they were more resistant to antimicrobials. Similarly, the phenotypic effects, such as higher D values and biofilm formation potentials, observed in the present study indicate a high metabolic response of these isolates, which also had greater resistance to PAA.

### 2.3. Large-Scale Genomic Association Between Presence of qac with stx1, stx2, or Both stx Genes from NCBI Pathogen Detection Database

To expand our findings beyond our sample set and also evaluate the impacts of *stx2*, we analyzed the global NCBI Pathogen Detection database for the presence of *qac* and *stx* genes. A total of 449,235 genomes were detected when only *E. coli*/*Shigella* species were selected. After the “Scientific name” filter was used and only *E. coli* strains were selected, the total number of genomes selected decreased to 393,216.

Subsequently, we filtered the genotypes to identify complete *qac* genes and their variants. A total of 104,016 gene matches were verified; however, 4743 genomes contained two or more *qac* variants within a single genome. As a result, 99,269 genomes (25.25% of the all-*E. coli* database) were identified as carrying one or more *qac* genes. Among these, the most frequently detected variants, in decreasing order, were as follows: *qacEdelta1* (85.93%; 85,302/99,269), *qacL* (16.26%; 16,143/99,269), *qacE* (1.61%; 1602/99,269), *qacG2* (0.82%; 816/99,269), *qacF* (0.12%; 121/99,269), and *qacC* (0.012%; 12/99,269). Also, less frequent variants included *qacH* (9), *qacG* (6), *qacK* (2), *qacB* (1), *qacR* (1), and *qacZ* (1), each representing less than 0.01% of the total.

Regarding the presence of *stx1* and its variants, 70,622 genomes were identified within the dataset, representing 17.96% of all *E. coli* genomes (70,622/393,216). However, only 3155 genomes (3.18%) contained both *stx1* and a *qac* gene, suggesting limited co-occurrence between these resistance and virulence determinants.

In addition, for the *stx2* toxin-encoding gene, our analysis identified its presence in 17.11% (67,262/393,216) of the *E. coli* genomes. When assessing the co-occurrence of *stx2* with a *qac* gene, we found that only 4.94% (4907/99,269) of the genomes carried both genes. In addition, when evaluating the simultaneous presence of both Shiga toxin-encoding genes (*stx1* and *stx2*) alongside a *qac* gene, we found that 8.12% (8062/99,269) of the genomes contained all three genes. Finally, the presence of *stx1*, *stx2,* or both was observed in 63.95% (251,347/393,216) of the available *E. coli* genomes in the database.

Therefore, to assess whether the distribution of *qac* genes was significantly associated with the presence or absence of *stx1* and *stx2*, we performed chi-square tests comparing *qac*-positive and *qac*-negative genomes across three groups: (i) *stx1*-positive and *stx1*-negative, (ii) *stx2*-positive and *stx2*-negative, and (iii) genomes carrying both *stx1* and *stx2* (Table 3).

Statistical analyses confirmed a significant association between *qac* genes and the absence of *stx1*, *stx2*, and both genes combined (*p* < 0.0001 for all comparisons; Table 3). These observed distributions suggest that the relationship between *qac* and *stx toxin* genes is not random.

Furthermore, to better understand this association and assess the relative risk between the presence of *qac* and Shiga toxin-encoding genes, we performed an Odds Ratio (OR) analysis. The results showed that the simultaneous presence of *stx1* and *qac* is least likely, with a *qac*-positive genome being approximately 89% less likely to carry *stx1* (OR = 0.11) compared to *stx1*-positive isolates without the *qac* gene (Table 3). This finding corroborates our newly sequenced dataset, which also demonstrated a weak association between *qac* and *stx1* genes.

Finally, the phi (φ) coefficient was used to measure the strength of an association between categorical variables. As shown in Table 3, the strongest observed association was between *qac* and *stx1* + *stx2* genes (φ = 0.25), followed by *stx1* and *qac* (φ = 0.22), and *stx2* and *qac* (φ = 0.19). Although the chi-square test statistically confirmed these associations, the phi values suggest only a weak-to-moderate correlation between these variables. However, considering the biological context and the large sample size analyzed (393,216 genomes), these results remain relevant. This observed pattern between *qac* and *stx* gene distribution suggests distinct evolutionary pressures, indicating that when *qac* is present, there may be selective pressure against *stx* genes, or vice versa, potentially favoring isolates with reduced virulence but increased sanitizer resistance. Furthermore, the statistical results from our broad database analyses reinforce our hypothesis of a delicate balance between virulence and resistance factors within the same isolate.

### 2.4. Analysis of Phage Insertion and Genome Synteny

After determining that *stx1* affected QAC resistance phenotypes, we performed a genetic alignment between the 24 newly sequenced strains using the position of *stx1* in the genome and the site of phage insertion in the DNA, as well as the presence of the QAC gene in strain S22. It has already been established [44] that the inclusion of *stx1* genes in *E. coli* was evolutionarily mediated by encoding phages, especially in their lysogenic cycle. Therefore, it is expected that the *stx1* gene would be in a region with low conservation and a high rate of genetic exchange. Furthermore, although the presence of genes associated with QAC resistance has always been attributed to plasmids and mobile genetic elements, some studies [45,46] have demonstrated the presence of these genes in chromosomal integrons integrated into bacterial DNA.

**Figure 5:**
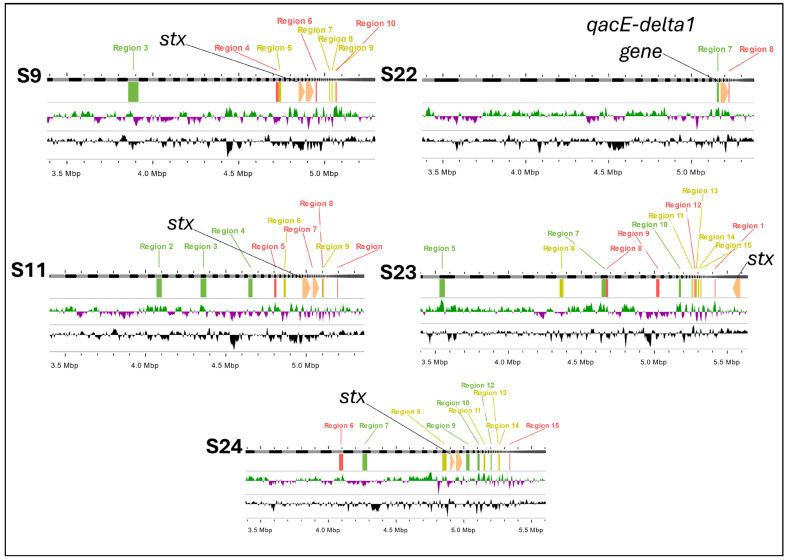
Phage and gene insertion sites in *E. coli* strains: *stx1* and *qac* gene localization. Legend: These genomic maps illustrate the insertion sites of phages and key genes (*stx1* and *qacE-delta1*) in five *E. coli* strains (S9, S11, S22, S23, and S24). Each panel represents the genome of a strain, highlighting phage insertion regions (labeled in green, red and purple) and the presence of the *stx1* gene (indicated in yellow) and *qacE-delta1* gene (indicated in black). Regions 1 through 15 mark specific phage insertion sites. The presence of the *stx1* gene correlates with multiple insertion regions, particularly in strains S9, S11, S23, and S24, while the *qacE-delta1* gene is uniquely identified in strain S22.

Thus, *stx1* and *qac* genes were found to be present at similar locations on the genome that were also categorized by their high genetic variability (Figure 5), suggesting that the relationship between *stx* presence and decreased QAC tolerance in the correlation and multiple correspondence analyses may be due to the high cost to the bacteria of maintaining both genes. Accordingly, *E. coli* likely substitutes genes at these loci based on which type of genetic information will provide the greatest fitness advantage.

Previously, we analyzed the impact of phage variability, and the presence of *stx* genes after the recultivation of *E. coli* [23]. We observed that the high potential for genomic rearrangement derived from the processes of the excision and insertion of phage-encoded regions makes the bacteria susceptible to genetic exchanges, probably influenced by environmental effects. These exchanges can facilitate the acquisition of new genes, including those that confer resistance to disinfectants, such as QACs.

A noteworthy point is that in most studies, resistance to QACs has been associated with genes located on plasmids, as is widely documented [10,11,47]. However, the inclusion of these genes in chromosomal DNA, rather than plasmid DNA, may possibly be mediated by mobile elements, such as chromosomal integrons. These elements are known to facilitate the incorporation of resistance genes into the bacterial chromosome [45,46].

In Figure 5, we present evidence that in strain S22, the QAC resistance gene was located on chromosomal DNA rather than on a plasmid. Genome filtering using the plasmidSPAdes tool confirmed the absence of plasmid-associated regions, indicating that QAC resistance for strain S22 was likely acquired through chromosomal integration. This finding suggests that phage- and mobile-element-mediated genotyping may allow the replacement or occupation of sites previously reserved for *stx* genes by QAC resistance genes, conferring an adaptive advantage to bacterial strains in environments where they are exposed to these disinfectants. These results reinforce the idea that although resistance to QACs is predominantly plasmid-borne, the incorporation of these genes into the bacterial chromosome is possible and may be associated with environmental selective pressures, as well as the genomic plasticity induced by mobile elements, such as integrons and transposons.

**Figure 6:**
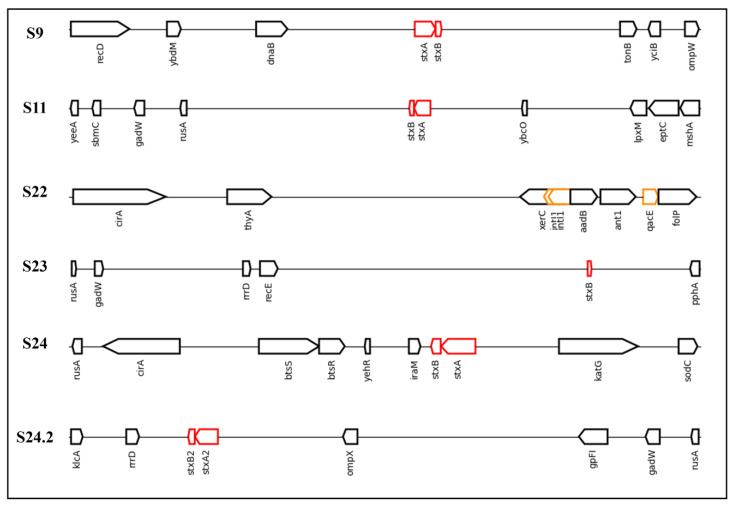
Linear DNA sequences found to possess *stx* or *qac* genes in the present study and the associated neighbor genes. Legend: The sequences analyzed in the present study were S9, S11, S22, S23, S24, and S24.2. The red rectangles represent Shiga toxin genes (*stx1* or *stx2*), while the yellow rectangles represent genes resistant to quaternary ammonium compounds (*qacEdelta1*) or the integron-integrase gene (*intI1*). This image was created using genoPlotR (version 0.8.11) in RStudio.

Finally, to analyze more precisely the presence of *stx* (S9, S11, S23 and S24) or *qacEdelta1* (S22) genes, we determined their inclusions in the genome and observed their arrangement in a linear image (Figure 6).

The results obtained demonstrate that *stx* genes included in the genomes were close to *rusA* in four of the five sequences that contained *stx* genes (S11, S23, S24, and S24.2). An important point is that the presence of *stx* close to the genes shown in Figure 6 is atypical. However, in a study performed by Yang et al. [48], the authors demonstrated that *rusA* shares 37.4% identity with prophage 75/02, an *stx*-encoding gene. Furthermore, *rusA* acts as a Holliday junction resolvase and has been described as a mediator of genetic recombination and cellular repair in the *E. coli* K12 prophage [49]. This indicates a region with high genetic variability.

Regarding the S22 sequence, which presents the QAC gene for resistance, although there is a clear difference between the neighboring genes and those that present a Shiga toxin gene, the sequence presented two insertions of *intI1*, a class 1 integron-integrase. These integrons assist in the insertion and excision of gene cassettes to confer resistance to bacteria [50]. Therefore, this presence reinforces the insertion of genes related to resistance to sanitizers in *E. coli* genomes and highlights possible intrinsic resistance conferred by this insertion. Furthermore, *intI1* has been proposed to be an indicator of antibiotic resistance in urban wastewater treatment plants [51]. An important point is that if we consider that *qacEdelta1* was by far the most common QAC gene present among the *E. coli* sequences from NCBI, this indicates a selective pressure for this gene and genetic adaptation of the bacteria. Further in silico studies should investigate the extent to which genes encoding resistance to QACs are integrated into bacterial genomes.

## 3. Materials and Methods

### 3.1. Strain Selection and DNA Extraction and Sequencing

Twenty-four *E. coli* strains were selected from meat samples, animal feces, and cattle production environments. The phenotypic profile of these strains was previously evaluated by Castro et al. [23] regarding their resistance profile to QACs and PAA using a minimum inhibitory concentration (MIC) methodology. Strain selection for WGS was conducted based on serogroups, as determined by PCR, and aimed to include low (6.25 ppm)-, intermediate (12.5 ppm)-, and high (25 ppm)-resistance profiles for QACs and PAA.

Therefore, the dataset comprised five O26 (three strains with intermediate QACs + PAA; two with intermediate QACs + high PAA), five O45 (one with low QACs + high PAA; four with intermediate QACs + PAA), four O103 (one with low QACs + high PAA; one with intermediate QACs + PAA; two with intermediate QACs + high PAA), two O111 (one with low QACs + intermediate PAA; one with low QACs + high PAA), four O121 (one with low QACs + intermediate PAA; one with intermediate QACs + low PAA; two with intermediate QACs + high PAA), and four O157 strains (one with low QACs + high PAA; one with intermediate QACs + PAA; two with intermediate QACs + high PAA). Also, it is important to highlight that the strains analyzed in this study belong to a DNA dataset that was not evaluated by Castro et al. [23]. All strain details are included in Table 1.

Subsequently, DNA extraction was performed using the DNeasy Blood & Tissue extraction kit (QIAGEN, Toronto, ON, Canada), following the manufacturer’s instructions to ensure DNA integrity and purity. After extraction, DNA quality and concentration were verified using the Qubit 3.0 (Life Technologies, Carlsbad, CA, USA) and, subsequently, the NanoDrop 1000 (Thermo Fisher Scientific, Waltham, MA, USA).

Whole-genome sequencing (WGS) was performed using the Illumina NovaSeq6000 platform (150 bp PE) with a minimum coverage of 300× to ensure data quality. Data quality was assessed using FASTQC (version 0.12.1), and sequences were processed and assembled using Shovill software (SPAdes, version 1.1.0). An additional check was performed to determine the presence of plasmids using plasdmidSPAdes (version 3.15.5). A linear visualization of the genomes was obtained using Matplotlib with the genoPlotR (version 0.8.11) package in biopython, with the data annotated by Prokka (version 1.14.6). All tools were used with the default settings. In addition, all sequences have been deposited in NCBI under BioProject PRJNA1166229.

### 3.2. Characterization of Serogroups, Virulence Genes, and Antimicrobial Resistance and Phylogenetic Analysis

The identification of serogroups was performed using two approaches: data from conventional PCR [23] and in silico typing based on WGS data, using Abricate (version 1.0.1) and the EcOH database. The concordance between the results of the two techniques was evaluated. Furthermore, other virulence genes were analyzed using ResFinder 4.6.0 (database: 22 March 2024) and DesinFinder (database: 31 May 2023) to detect genes related to resistance to sanitizers and antibiotics.

Phylogenetic analysis was performed on annotated WGS data using Prokka (version 1.14.6). The *gff*-format files were used to perform both core-genome (minimum 95% identity and 90% of isolates having the gene needed to be classified as core) and accessory gene alignment using Roary (version 3.13.0). The phylogenetic tree was constructed using the core-genome alignment output in Newick format from Roary. Furthermore, the phylogenetic tree was constructed using IQ-tree (version 2.3.6) and an ultrafast bootstrap parameter of 1000 replicates. Images were edited using the website ITOL: https://itol.embl.de/ (accessed on 5 February 2025).

For multiple correspondence analyses, we used accessory genes obtained from Roary output to filter for genes of interest for inclusion in correlation. For this filtering, we selected genes present in less than 50% of all sequences to enhance the chances of elucidating heterogeneous MIC values. Also, we screened the genes related to acidity tolerance or antimicrobial resistance from these accessory genes. Therefore, the genes used were *aph(3)-Ib*, *aph(6)-Id*, *blatem-1b*, sul, *tet(a)*, *tet(b)*, *tet(c)*, *tet(m)*, *flor*, *dfra14*, *colrnaI*, *gadA*, *gadB*, *emrE*, *cbtA*, and *stx*. In addition, the phenotypic results included QAC and PAA MIC values, the *D* 60 °C value, and the season of isolation.

Finally, the investigation of the contribution of phages resident in the genome was performed using the Phastest.ca website, employing deep mode and disabling the option to use pre-computed results. For this purpose, the sequences used were first aligned using the ClustalW alignment tool (version 2.1), using the IUB method to generate the DNA matrix for a complete alignment. With this methodology, we ensured that the phage insertion sites obeyed a specific order and could be compared through the insertion sites of the previously characterized *stx* and *qacE-delta 1* genes.

### 3.3. Comparison of Presence of qac with stx1, stx2, or stx1 and stx2 Using the NCBI Pathogen Detection Database

To expand our observations to a global level, we used the NCBI Pathogen Detection system: https://www.ncbi.nlm.nih.gov/pathogens/ (accessed on 4 February 2025). We selected *E. coli*/*Shigella* as the species. Subsequently, we applied the “Scientific name” filter and filtered only for those described as *E. coli*. After this, using the “Stress genotypes” filter, we filtered for sequences that included the complete presence of QAC genes and variants. Using the set of sequences mentioned above, we applied the “Virulence genotypes” filter and selected all genomes that presented at least one complete gene for *stx1*, *stx2*, or *stx1* and *stx2*. Finally, we also compiled the values for the presence of genomes with the inclusion of the *stx1*, *stx2*, or *stx1* and *stx2* genes or variants without the insertion of any *qac* gene.

### 3.4. Statistical Analysis of Data

Statistical analyses were conducted using R^®^ version 4.1.3 (10 March 2022) (R Foundation for Statistical Computing, Vienna, Austria) [52] to examine correlations and multiple correspondences between the presence of virulence and resistance genes and phenotypic data, such as MIC values for QACs and PAA. Spearman’s correlation coefficient was applied to assess the relationship between the presence of specific genes, such as *stx1* and *cbtA*, and the MIC values obtained.

In addition, a multivariate multiple correspondence analysis (MCA) [53,54] was performed to explore the associations between the presence of resistance genes, and virulence, phenotypic, and environmental variables, such as the sanitizer resistance, biofilm formation capacity, and seasonality of the samples. The MCA results were represented graphically, allowing a clear visualization of the contributions of each variable in the different dimensions analyzed. The R^®^ libraries used to perform MCA were “FactoMiner” [55] and “factoextra” [56].

Finally, to assess the association between the presence of QAC and the *stx1*, *stx2*, or *stx1* and *stx2* genes in *E. coli* genomes from the NCBI Pathogen Detection database, we applied three chi-square (χ^2^) tests using a 2 × 2 contingency table (1. *qac*-positive/*qac*-negative and *stx1*-positive/*stx1*-negative; 2. *qac*-positive/*qac*-negative and *stx2*-positive/*stx2*-negative; 3. *qac*-positive/*qac*-negative and *stx1*- and *stx2*-positive/*stx1*- and *stx2*-negative). Also, the Odds Ratio (OR) with a 95% confidence interval and the phi (φ) coefficients were utilized to determine the strength of an association. All analyses were performed using R^®^ version 4.1.3 (10 March 2022).

## 4. Conclusions

The present study explored the differences between the resistance profiles of twenty-four *E. coli* strains to two sanitizers frequently used in the food industry (quaternary ammonium and peracetic acid). Our initial findings revealed a negative correlation between the presence of *stx1* and resistance to QAC-based sanitizer, suggesting that strains carrying *stx1* tend to be more sensitive to QACs. In contrast, the presence of the *cbtA* gene (inhibiting cell elongation and division function) showed a positive correlation with resistance to PAA acid. Though multiple correspondence analysis, these data reinforced that the presence of the *stx1* gene was associated with a sensitive QAC profile. This information led us to expand our investigation to the interaction between *qac* and the *stx1* + *stx2* genes. For this purpose, we conducted a global assessment, analyzing 393,216 genomes from the NCBI Pathogen Detection database. This analysis confirmed a statistically significant pattern of low co-occurrence between *qac* genes and *stx1*, *stx2*, or both genes, again corroborating our initial results.

Moreover, synteny and phage inclusion analysis indicated that the *stx1* and *qac*E-delta1 genes may occupy similar positions in the genome in non-conserved regions, suggesting that genomic rearrangements may be involved in the exchange of these genes, with the choice being “either/or” instead of “and”. This observation aligns with our hypothesis that strains with increased pathogenicity tend to lack resistance to sanitizers. For instance, in a biofilm environment, minimally pathogenic or non-pathogenic guardian bacteria in the outermost layers may act as a shield, while more pathogenic strains which are susceptible to sanitizers remain protected in the deeper layers, evading the effects of these agents. Further studies are required to investigate the application of sanitizers in mixed biofilms (non-pathogenic and pathogenic *E. coli*) and compare survival rates between groups. In addition, we highlight that while the present study provides valuable insights into sanitizer exposure and virulence genome arrangements, further validation is needed. Specifically, the modification of the genome of a generic strain of *E. coli* using *stx* and *qac* genes in combination with sanitizer exposure would be necessary to fully understand the behavior and underlying mechanisms of this genetic arrangement.

Finally, serogroups O26, O45, and O157 exhibited a multidrug resistance profile compared to O111, O121, and O103, reinforcing the relevance of additional studies to better understand the interactions among serogroup and antimicrobial resistance dynamics.

## Figures and Tables

**Table 1:** Strains used in this study and their quaternary ammonium compound (QAC) and peracetic acid (PAA) resistance.

Strain	Serogroup by PCR *	Serotype by WGS	STEC or Tolerance Gene Presence	Ammonium Quaternary Resistance (ppm) *	Peracetic Acid Resistance (ppm) *
S1	O45	O45:H19	Negative	12.5	12.5
S2	O26	O26	Negative	12.5	25
S3	O103	O103:H2	Negative	6.25	25
S4	O157	O154:H55 **	Negative	12.5	25
S5	O45	O45:H9	Negative	12.5	12.5
S6	O45	O45:H4	Negative	6.25	25
S7	O103	O103:H2	Negative	12.5	12.5
S8	O103	O103:H2	Negative	12.5	25
S9	O111	O111:H8	*stx1*-Positive	6.25	12.5
S10	O121	O121:H46	Negative	6.25	12.5
S11	O111	O111:H8	*stx1*-Positive	6.25	25
S12	O26	O26	Negative	12.5	12.5
S13	O157	O157:H29	Negative	12.5	25
S14	O26	O131:H26 **	Negative	12.5	12.5
S15	O26	O26:H11	Negative	12.5	12.5
S16	O103	O103:H21	Negative	12.5	25
S17	O45	O45:H4	Negative	12.5	12.5
S18	O121	O121:H46	Negative	12.5	6.25
S19	O45	O45:H4	Negative	12.5	12.5
S20	O121	O121:H46	Negative	12.5	25
S21	O121	O121:H7	Negative	12.5	25
S22	O26	O17:H18 **	Negative, *qacE*-Positive	12.5	25
S23	O157	O157:H7	*stx1*-Positive	6.25	25
S24	O157	O157:H7	*stx1*- *and stx2*-Positive	12.5	12.5

Legend: * (Castro et al. [23]); ** difference between PCR and WGS.

**Table 2:** Correlation between genes and sanitizers.

Genes	Sanitizer	Spearman’s Correlation Coefficient
*stx*	Quaternary Ammonium	−0.694
*cbtA*	Peracetic Acid	0.691

**Table 3:** Analysis of genomes present in the NCBI Pathogen Detection database for the co-occurrence of *qac* and *stx* genes.

*qac* and *stx1* Genes			
Variables	*stx1* Gene +	*stx1* Gene −	Total
*qac* Gene +	3155 (3.17%)	96,114 (96.83%)	99,269
*qac* Gene −	67,467 (22.95%)	226,480 (77.05%)	293,947
Total	70,622 (17.96%)	322,594 (82.04%)	393,216
Statistical Analysis			
Chi-Square (χ^2^, df = 1)	19.67; *p* < 0.0001		
Odds Ratio (IC 95%)	0.11 (0.106–0.114)		
Phi (φ) Coefficient	0.22		
***qac*** **and *stx2* Genes**			
Variables	*stx2* gene +	*stx2* gene −	Total
*qac* Gene +	4907 (4.94%)	94,362 (95.06%)	99,269
*qac* Gene −	62,355 (21.21%)	231,592 (78.79%)	293,947
Total	67,262 (17.10%)	325,954 (82.90%)	393,216
Statistical Analysis			
Chi-Square (χ^2^, df = 1)	13.85; *p* < 0.0001		
Odds Ratio (IC 95%)	0.19 (0.184–0.196)		
Phi (φ) Coefficient	0.19		
***qac*** **and *stx1* and *stx2* Genes**			
Variables	*stx1* and *stx2* gene +	*stx1* gene and *stx2* −	Total
*qac* Gene +	8062 (8.12%)	91,207 (91.88%)	99,269
*qac* Gene −	105,401 (35.86%)	188,546 (64.14%)	293,947
Total	113,463 (28.86%)	279,753 (71.14%)	393,216
Statistical Analysis			
Chi-Square (χ^2^, df = 1)	24.74; *p* < 0.0001		
Odds Ratio (IC 95%)	0.17 (0.166–0.174)		
Phi (φ) Coefficient	0.25		

## Data Availability

All sequences have been deposited in NCBI under BioProject PRJNA1166229.
